# Comparative Screening of Mexican, Rwandan and Commercial Entomopathogenic Nematodes to Be Used against Invasive Fall Armyworm, *Spodoptera frugiperda*

**DOI:** 10.3390/insects13020205

**Published:** 2022-02-16

**Authors:** Patrick Fallet, Lara De Gianni, Ricardo A. R. Machado, Pamela Bruno, Julio S. Bernal, Patrick Karangwa, Joelle Kajuga, Bancy Waweru, Didace Bazagwira, Thomas Degen, Stefan Toepfer, Ted C. J. Turlings

**Affiliations:** 1 Laboratory of Fundamental and Applied Research in Chemical Ecology, Institute of Biology, University of Neuchâtel, Rue Emile-Argand 11, CH-2000 Neuchâtel, Switzerland; patrick.fallet@unine.ch (P.F.); ldegia@hotmail.ch (L.D.G.); pamela.bruno@agr.uni-goettingen.de (P.B.); thomas.degen@unine.ch (T.D.); 2 CABI, Rue des Grillons 1, CH-2800 Delémont, Switzerland; s.toepfer@cabi.org; 3 Experimental Biology Research Group, Institute of Biology, University of Neuchâtel, Rue Emile-Argand 11, CH-2000 Neuchâtel, Switzerland; ricardo.machado@unine.ch; 4 Department of Entomology, Texas A&M University, College Station, TX 77840, USA; juliobernal@tamu.edu; 5 Rwanda Agriculture and Animal Resources Development Board (RAB), KG 569 Street, Kigali 5016, Rwanda; patrick.karangwa@rab.gov.rw (P.K.); joelle.kajuga@rab.gov.rw (J.K.); bancywaweru@yahoo.com (B.W.); bazagwiradidace@gmail.com (D.B.); 6 MARA-CABI Joint Laboratory for Biosafety, Institute of Plant Protection, Chinese Academy of Agricultural Sciences, Beijing 100193, China

**Keywords:** biological control, integrated pest management, East Africa, maize, invasive species, food security

## Abstract

**Simple Summary:**

The fall armyworm is a devastating insect pest of maize that has recently spread from the Americas to Africa and Asia. Synthetic insecticides are currently being used excessively to fight this pest. Safe, effective and more sustainable alternatives are urgently needed. We explore the use of beneficial entomopathogenic nematodes to control the fall armyworm. These tiny soil-born roundworms are lethal parasites of insects, including caterpillars such as the fall armyworm. We tested forty nematode strains from either the native range of the fall armyworm (Mexico), or the area of invasion (Rwanda), and commercial strains. We found that certain strains of local nematodes from the area of invasion can be as effective in infecting and killing fall armyworm as commercial strains or those from the armyworm’s native range. These findings will aid the development of locally acceptable and effective biological control products.

**Abstract:**

The fall armyworm (FAW), *Spodoptera frugiperda* Smith (Lepidoptera: Noctuidae) is an important pest of maize originating from the Americas. It recently invaded Africa and Asia, where it causes severe yield losses to maize. To fight this pest, tremendous quantities of synthetic insecticides are being used. As a safe and sustainable alternative, we explore the possibility to control FAW with entomopathogenic nematodes (EPN). We tested in the laboratory whether local EPNs, isolated in the invasive range of FAW, are as effective as EPNs from FAW native range or as commercially available EPNs. This work compared the virulence, killing speed and propagation capability of low doses of forty EPN strains, representing twelve species, after placing them with second-, third- and sixth-instar caterpillars as well as pupae. EPN isolated in the invasive range of FAW (Rwanda) were found to be as effective as commercial and EPNs from the native range of FAW (Mexico) at killing FAW caterpillars. In particular, the Rwandan *Steinernema carpocapsae* strain RW14-G-R3a-2 caused rapid 100% mortality of second- and third-instar and close to 75% of sixth-instar FAW caterpillars. EPN strains and concentrations used in this study were not effective in killing FAW pupae. Virulence varied greatly among EPN strains, underlining the importance of thorough EPN screenings. These findings will facilitate the development of local EPN-based biological control products for sustainable and environmentally friendly control of FAW in East Africa and beyond.

## 1. Introduction

The fall armyworm (FAW), *Spodoptera frugiperda* Smith (Lepidoptera: Noctuidae) is a major pest of maize originating from the tropical and subtropical regions of the Americas [1]. FAW is polyphagous, but prefers grasses, particularly maize, sorghum and rice [1]. In early 2016, FAW was reported in West Africa and rapidly spread across the continent and then further into Asia [2,3,4,5]. On both continents, it causes tremendous crop damages and yield losses [5,6,7,8,9]. To mitigate the impact of FAW, governments launched emergency programs and subsidized synthetic insecticides, which quickly became the backbone of FAW control in the invasive range [10,11,12]. Because of the environmental harm and health risks caused by these measures, safe, sustainable and effective alternative FAW control strategies are urgently needed [5].

Biological control of pest insects using entomopathogenic nematodes (EPN) has been implemented with success for several decades, particularly against belowground pests [13]. EPN are tiny soil-dwelling roundworms that are naturally present in soils worldwide [14]. They can be isolated from soil samples, mass produced and then applied to control pest insects. Importantly, they are safe for farmers or consumers, and cause virtually no harm to the environment [15,16]. EPNs can be particularly virulent to lepidopteran caterpillars, including FAW, but the virulence against a target insect greatly varies between EPN species and strains [17,18,19,20,21,22,23]. Hence, EPN screenings are necessary to identify promising EPNs for specific biological control programs [20].

We have launched a project to use EPNs against FAW caterpillars, by applying EPN formulations into the whorl of maize plants. We intend to use locally isolated EPNs to avoid introducing foreign organisms. This is important, as it is increasingly evident that EPN species are not always correctly identified [24,25]. Misdiagnosing closely related EPN species increases the risk of accidently introducing non-native EPNs in a targeted region, as has been reported previously [26].

The aim of the current study was to compare the virulence and killing speed of different species and strains of EPN to identify promising EPN candidates for biological control of FAW in East Africa. We used EPNs from different areas of origin that were either locally isolated in Rwanda and in Mexico, or that were obtained from commercial sources. We hypothesized that EPN strains from Mexico, where FAW is native, may have evolved higher virulence than EPNs from Rwanda, in the invasive range [27], while commercial strains are expected to be the most virulent, as they have been selected for this trait.

We compared forty strains of EPN, representing twelve species, in three steps. In the first step, we compared the virulence and the killing speed of twenty-nine Mexican EPN strains, representing five species, against third-instar FAW caterpillars. The five most virulent Mexican strains (four species) were selected for the second step, in which they were compared with five Rwandan (three species) and six commercial strains (six species). In the third step, the five most promising EPN from the second step (one Mexican, two Rwandan and two commercial EPN) were tested at three concentrations (5, 25, 125 EPN per FAW) against second-, third- and sixth-instar caterpillars as well as against pupae of FAW. In addition, we evaluated reproductive output of each of these EPN strains in FAW caterpillars. The overall aim was to determine the level of virulence of specific EPN strains against FAW and to lay the basis for their development as biological control agents. An additional objective was to clarify whether such efforts should focus on EPN from the area-of-origin of the pest, or whether commercial EPN or EPN native to the target region are just as effective.

## 2. Materials and Methods

### 2.1. Origin and Handling of the Fall Armyworm and Nematodes

*Spodoptera frugiperda* (FAW) caterpillars were reared on artificial diet (Beet Armyworm Diet, Frontier Scientific, Newark, NJ, USA) under quarantine conditions (OFEV permit A140502) at the University of Neuchâtel, Switzerland.

Forty entomopathogenic nematodes strains, representing twelve species, were used in this study (Table 1). They were either collected from soil samples in Mexico [28], Rwanda [29,30] or obtained from commercial sources (e-nema GmbH, Schwentinental, Germany; Guangdong Academy of Sciences, Guangdong, China). EPN were reared in vivo on last-instar *Galleria mellonella* L. (Lepidoptera: Pyralidae). They were stored at 12 °C in the dark and used in experiments within ten days post emergence.

### 2.2. Step 1: Screening of Mexican Entomopathogenic Nematodes against the Fall Armyworm

The virulence and killing speed of the 29 Mexican EPN strains (five species) were evaluated on third-instar FAW caterpillars. One strain of *Heterorhabditis atacamensis*, twenty-one of *Heterorhabditis bacteriophora,* three of *Heterorhabditis zacatecana* (newly described by [25]), three of *Heterorhabditis mexicana* and one of *Steinernema riobrave* were used (Table 1). The virulence of each Mexican EPN strain was compared to the virulence of the commercial EPN *Steinernema carpocapsae* strain All, which was used as the positive control. In total, eighteen to twenty-four caterpillars were exposed to each EPN strain in three independent experiments.

Briefly, caterpillars were weighed and then individually placed in an arena (4 cm long × 4 cm wide × 2.5 cm height) containing a filter paper folded on the sides (5.5 cm in diameter) and artificial diet (ca. 1 cm^3^). The average weight (± SD) of the third-instar caterpillars was 10.6 ± 1.7 mg (n = 760). Ten infective juveniles (IJs) were applied in 400 µL of water in each arena (10 IJs per caterpillar). As control, only water was applied. Arenas were stored in the dark at 24 ± 1 °C, and caterpillar mortality was recorded daily for seven days. Mortality was assessed and corrected using Abbott’s formula [31] as follows:

$$\frac{\mathrm{survival}\;\mathrm{in}\;\mathrm{the}\;\mathrm{control}\;-\;\mathrm{survival}\;\mathrm{in}\;\mathrm{the}\;\mathrm{treatment}}{\mathrm{survival}\;\mathrm{in}\;\mathrm{the}\;\mathrm{control}}\;\times \;100$$

The killing speed of the most virulent EPNs, which were selected to be used in the second experiment, was compared amongst one another. These were *H. bacteriophora* (two strains: MEX-17, MEX-35), *H. mexicana* (two strains: MEX-47, MEX-25), *H. zacatecana* (MEX-41) as well as *S. riobrave* (MEX-15).

To evaluate the reproduction success of the EPNs (= propagation), cadavers were collected and weighed daily. They were then individually placed in nematode traps [32] for three weeks. The IJs that had emerged were then counted under a stereoscopic microscope in five replicates per cadaver. Because the size of the cadavers differed greatly (as some nematodes killed caterpillars faster than others), and with it the number of IJs that could emerge from each cadaver, the reproduction was expressed as the number of IJs per mg of the cadaver (IJs ∗ mg^−1^). The reproduction of different EPN was not compared statistically, as different EPN species differ considerably in the number of offspring per se. It was only used to determine whether each EPN strain could reproduce in FAW and to determine how many IJs can emerge from a cadaver. The reproduction of strains that killed fewer than five caterpillars was not evaluated.

### 2.3. Step 2: Comparing the Virulence of Mexican, Rwandan and Commercial Entomopathogenic Nematodes against the Fall Armyworm

The virulence and killing speed of five Mexican (highly efficient EPNs as per previous screening), five Rwandan, and six commercial EPN were evaluated using third-instar FAW caterpillars. The experiment was conducted as described above with each individual caterpillar exposed to ten IJs of each EPN strain. The average weight (± SD) of the caterpillars was 10.3 ± 1.3 mg (n = 408). The Mexican EPN that were selected based on the previous screening results, included two strains of *H. mexicana* (MEX-25, MEX-47), two of *H. bacteriophora* (MEX-17, MEX-35), one of *H. zacatecana* (MEX-41) and one of *S. riobrave* (MEX-15). The Rwandan EPN were three strains of *Heterorhabditis ruandica* (RW14-N-C4a, Rw18_M-Hr1a, Rw18_M-Hr1b), one of *S. carpocapsae* (RW14-G-R3a-2) and one undescribed species *Steinernema* sp. (closely related to *Steinernema feltiae*; RW14-M-C2b-1). The commercial EPN were *S. carpocapsae* All (Nemastar^®^), *H. bacteriophora* (Dianem^®^), *Steinernema abbasi*, *Steinernema feltiae* (Nemaplus^®^), *Heterorhabditis beicherriana* H06 (HR-HB^®^) and *Heterorhabditis indica* LN2 (HR-HI^®^). In total, sixteen to twenty-four caterpillars were exposed to each EPN strain in three independent experiments.

The virulence of each EPN strain was compared to the virulence of the commercial EPN *S. carpocapsae* All, which was used as positive control. The killing speed of a few highly effective strains, which were selected to be used in further experimentation, were compared amongst one another. These EPNs were among the most virulent and fast killing strains originating from Mexico, Rwanda or commercial sources: *S. carpocapsae* strain All, *S. abbasi, S. carpocapsae* RW14-G-R3a-2, *H. ruandica* Rw18_M-Hr1a and *H. zacatecana* MEX-41. *H. zacatecana* MEX-41 was not the most effective Mexican strain, but it was selected for experiment three because it is closely related to *H. ruandica* as well as to *Heterorhabditis bacteriophora*. The latter is commonly used in commercial products. The reproduction success of each EPN was evaluated as described above.

### 2.4. Step 3: Assessing the Dose-Dependent Effectiveness of the Most Promising Entomopathogenic Nematodes against Different Stages of the Fall Armyworm

Five of the most promising EPN strains were selected, from the preceding two steps, with at least one strain from each origin (Mexican, Rwandan or commercial). The Rwandan EPN were *S. carpocapsae* RW14-G-R3a-2 and *H. ruandica* Rw18_M-Hr1a (formerly considered to be *H. bacteriophora* [25,30]). The Mexican EPN was *H. zacatecana* MEX-41. The commercial EPN were *S. carpocapsae* All (Nemastar^®^) and *S. abbasi.* The virulence and the killing speed of these strains were tested against second-, third- and sixth-instar FAW caterpillars as well as against FAW pupae. The experiment was conducted as described above, but using three concentrations of IJs: 5, 25 and 125 IJs per arena (individual caterpillar or pupa). To ensure that pupal mortality was not caused by EPN killing emerging adults (when the adult cracks open its pupa), five days post inoculation, pupae were plunged for a few seconds in bleach (0.5%), rinsed twice with water and transferred to clean arenas without EPN. The average weight (±SD) of the caterpillars was 1.2 ± 0.1 mg (n = 413), 6.2 ± 2.6 mg (n = 416), 447 ± 151 mg (n = 410) for second-, third- and sixth-instar, respectively. It was 252 ± 38 mg (n = 400) for the pupae. The virulence and the killing speed were compared amongst the EPN strains. Due to the low mortality of pupae, the killing speed of EPN was not evaluated for this developmental stage. In total, nineteen to thirty-one FAW individuals were exposed to each treatment (strains and concentrations) in three independent experiments.

The reproductive success of each EPN on FAW caterpillars was evaluated as described above. Since too few pupae died, reproductivity was not evaluated for the pupal stage.

### 2.5. Data Analyses

Statistical analyses were performed using R version 4.1.0 (R Core Team 2021).

Due to the complete separation of the data (100% or 0% mortality in some treatments), EPN virulence was analyzed using Bayesian generalized linear models (“arm” package [33]) with a binomial error distribution. In steps one and two, mortality of caterpillars was used as the response variable, while treatment and replication (each experiment was replicated three times) were used as fixed factors. The virulence of each strain was then compared to the virulence of the commercial EPN *S. carpocapsae* All, using many-to-one comparisons (“emmeans” package [34]) corrected for false discovery with the Benjamini and Hochberg method [35]. In step three, mortality of caterpillars was used as the response variable, while treatment, concentration (number of IJs used), FAW stage, and replication (each experiment was replicated three times) were used as fixed factors. Interaction effects between fixed factors that were not significant were removed from the model. Virulence was compared amongst EPN strains within each pair of concentration and insect stage using Bayesian generalized linear models with a binomial error distribution, followed by multiple comparisons (“emmeans” package [34]) corrected for false discovery using the Benjamini and Hochberg method [35].

EPN killing speed was analyzed using Cox proportional hazards regression models (“survival” package [36]) followed by multiple comparisons of survival curves (“survminer” package [37]) corrected for false discovery with the Benjamini and Hochberg method [35]. In steps one and two, the survival of FAW caterpillars was used as the response variable, while treatment and replication (each experiment was replicated three times) were used as fixed factors. Only the most effective strains (see Methods above) were compared in the multiple comparisons. In step three, the survival of FAW caterpillars or pupae was used as the response variable, while treatment, concentration (number of IJs used), FAW stage and replication (each experiment was replicated three times) were used as fixed factors. Interaction effects between fixed factors that were not significant were removed from the model. Multiple comparisons of survival curves were performed within each pair of concentration and insect stage.

In steps one and two, statistical analyses were not performed on EPN reproduction, as EPN species produce different numbers of offspring per se. EPN reproduction was only used to determine whether each EPN strain could reproduce on FAW and to evaluate the number of IJs that could emerge from a cadaver. In step three, the effects of both inoculation dose as well as development stage of the caterpillars at death on EPN propagation were evaluated using zero-inflated regression models for count data with a Poisson error distribution (“pscl” package [38]).

## 3. Results

### 3.1. Step 1: Screening of Mexican Entomopathogenic Nematodes against the Fall Armyworm

**Figure 1:**
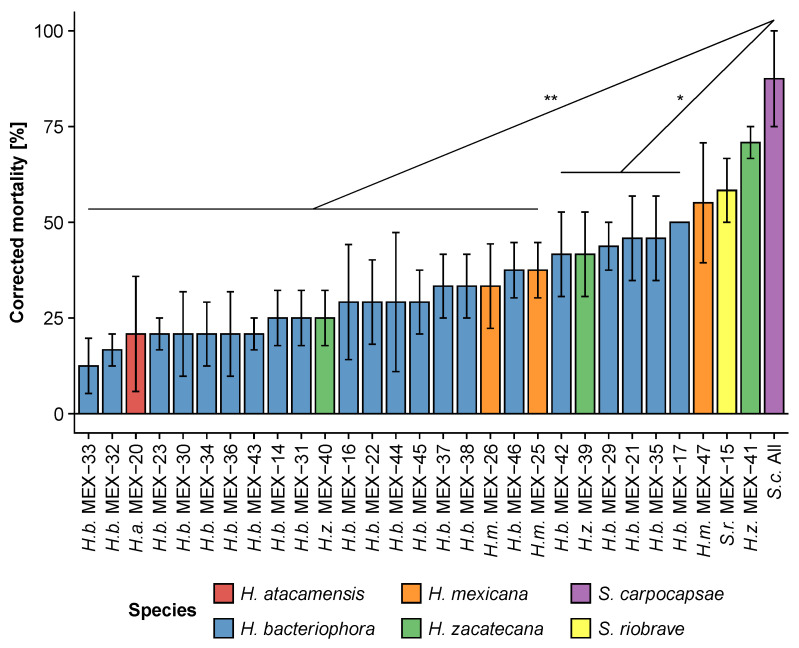
Virulence (mean ± SE) of Mexican entomopathogenic nematode strains (MEX) on third-instar *Spodoptera frugiperda* caterpillars in small arena laboratory bioassays. Mortality was evaluated seven days post inoculation with ten infective juvenile nematodes per caterpillar. The virulence of each Mexican strain was compared to the commercial *Steinernema carpocapsae* strain All (positive control). Stars (*) indicate significant differences obtained from many-to-one comparisons corrected for false discovery using the Benjamini and Hochberg method (no star: non-significant, *: *p* < 0.05, **: *p* < 0.01).

All 29 Mexican EPN strains were able to infect and kill FAW caterpillars. The application of ten infective juveniles significantly reduced third-instar caterpillar survival (χ^2^_(29)_ = 76, *p* < 0.001; Figure 1). The positive control, the commercial *Steinernema carpocapsae* All, was among the most virulent strains and caused 88 ± 12.5% (mean ± SE) mortality. Three of the Mexican strains were as virulent as *S. carpocapsae* All (*p* > 0.05): *Heterorhabditis zacatecana* MEX-41 (71 ± 4.2%), *Steinernema riobrave* MEX-15 (58 ± 8.3%) and *Heterorhabditis mexicana* MEX-47 (55 ± 12.6%; *p* > 0.05). All the other Mexican strains were less virulent than *S. carpocapsae* All (*p* < 0.05). Notably, EPN virulence varied greatly within the same species. For instance, *H. bacteriophora* MEX-17 was four times more virulent than *H. bacteriophora* MEX-33 (Figure 1).

**Figure 2:**
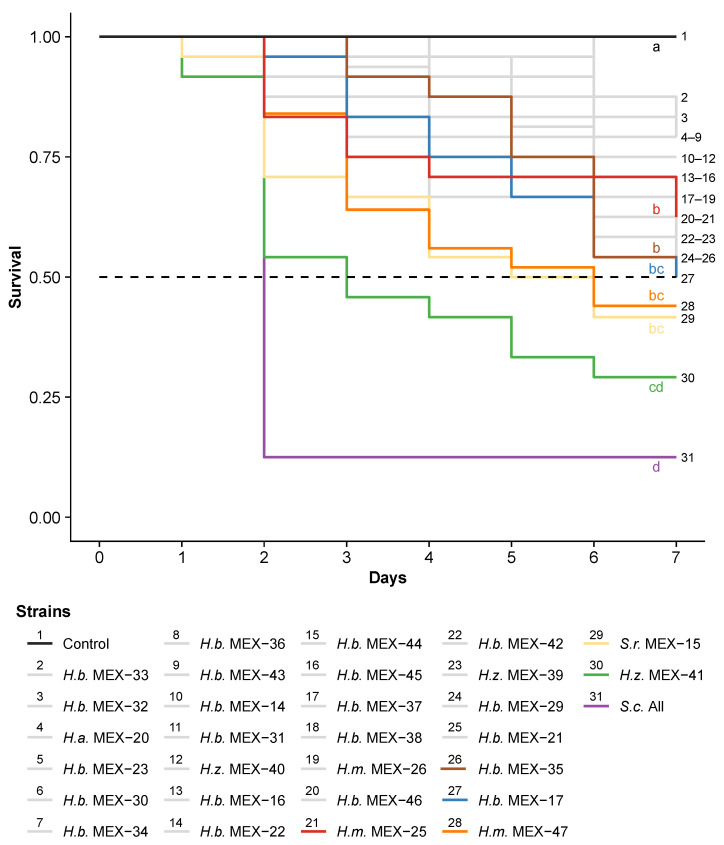
Killing speed of Mexican nematodes strains (MEX) of *Heterorhabditis bacteriophora* (H.b.), *H. mexicana* (H.m.), *H. zacatecana* (H.z.) and *Steinernema riobravae* (S.r.) when placed with third-instar *Spodoptera frugiperda* caterpillars in small arenas. Mortality was evaluated over seven days post inoculation with ten infective juvenile nematodes per caterpillar. The commercial nematode *Steinernema carpocapsae* (S.c.) All was used as positive control. To facilitate readability, only a few highly virulent strains, which were selected for further experimentation, are highlighted in colors. Less virulent strains are represented in grey. The dotted horizontal line represents 50% survival. Letters indicate significant differences (*p* < 0.05) obtained from multiple comparisons corrected for false discovery using the Benjamini and Hochberg method.

EPN species and strains differed in their killing speed of third-instar FAW caterpillars (χ^2^_(29)_ = 51, *p* = 0.008; Figure 2). The commercial *S. carpocapsae* All as well as the Mexican *H. zacatecana* MEX-41 were the fastest in killing caterpillars with an LT_50_ of two and three days, respectively. The other strains needed more time than *S. carpocapsae* All to kill FAW caterpillars (*p* < 0.05).

All Mexican strains successfully reproduced in FAW caterpillars (Table S1).

### 3.2. Step 2: Comparing the Virulence of Mexican, Rwandan and Commercial Entomopathogenic Nematodes against the Fall Armyworm

**Figure 3:**
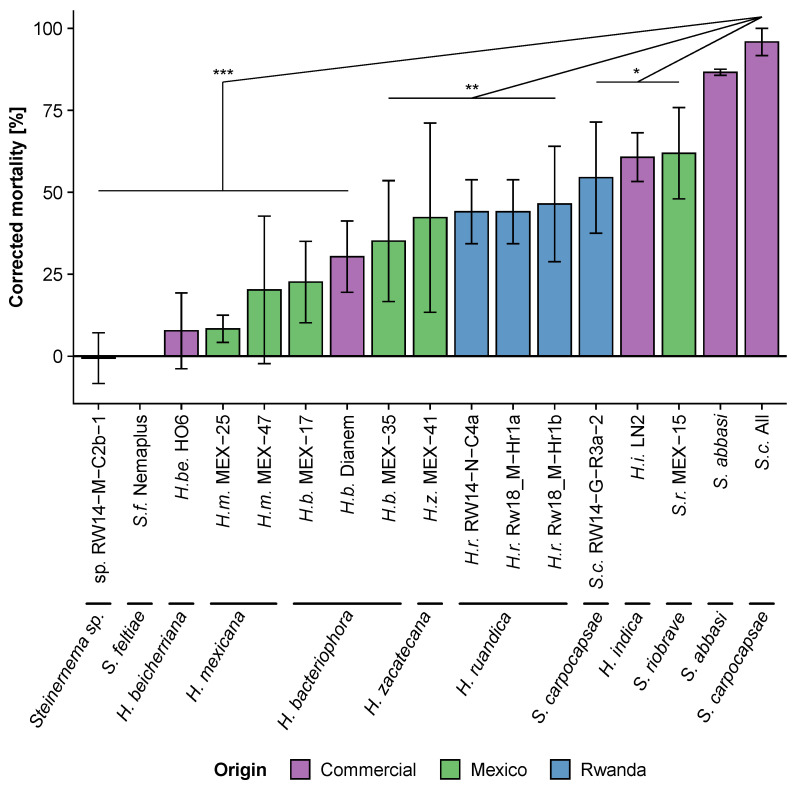
Virulence (mean ± SE) of Mexican, Rwandan and commercial entomopathogenic nematode strains when placed with third-instar *Spodoptera frugiperda* caterpillars in small arenas. The mortality of individual caterpillars was evaluated seven days post inoculation with ten infective juvenile nematodes per caterpillar. The virulence of each strain was compared to the virulence of the commercial *Steinernema carpocapsae* strain All, which served as the positive control. Stars (*) indicate significant differences obtained from many-to-one comparisons corrected for false discovery using the Benjamini and Hochberg method (no star: non-significant, *: *p* < 0.05, **: *p* < 0.01, *** and *p* < 0.001).

EPNs significantly reduced third-instar FAW caterpillar survival (χ^2^_(16)_ = 122, *p* < 0.001; Figure 3). The commercial *S. carpocapsae* strain All and *Steinernema abbasi* appeared to be the most lethal (*S. carpocapsae* All vs. *Steinernema abbasi*: *p* > 0.05) and killed 96 ± 4.2% and 87 ± 0.9% (mean ± SE) of caterpillars, respectively. The Rwandan *S. carpocapsae* (RW14-G-R3a-2; 54 ± 17%), the Mexican *S. riobrave* (MEX-15; 54 ± 17%), as well as the commercial *H. indica* (LN2; 61 ± 7.4%) were nearly as virulent as *S. carpocapsae* All (0.01 < *p* < 0.05). The other strains were found to be less virulent (*p* < 0.01).

**Figure 4:**
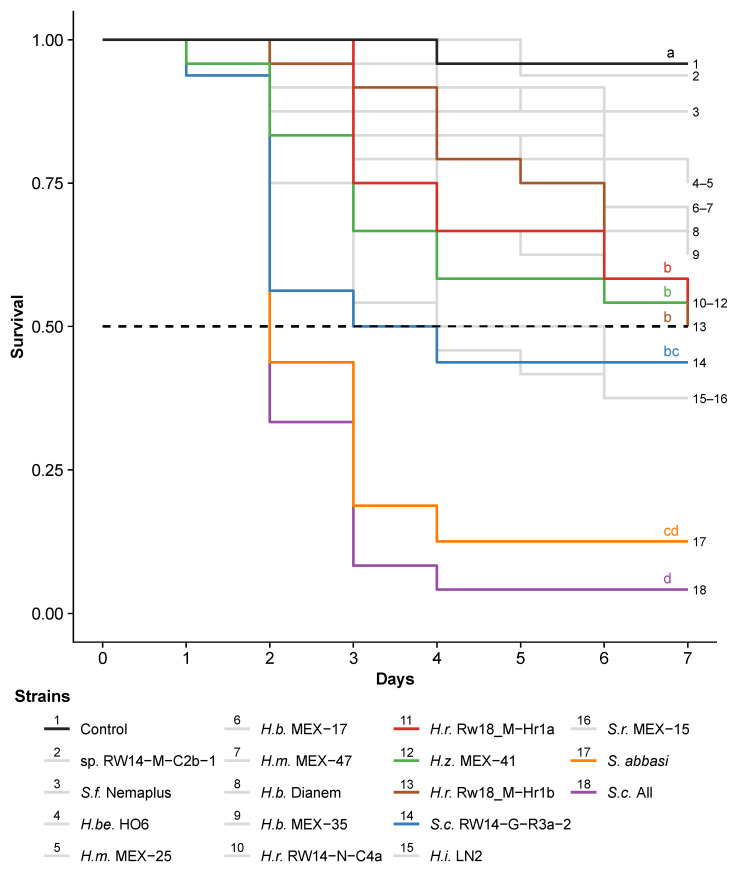
Killing speed of Mexican, Rwandan and commercial nematodes strains on third-instar *Spodoptera frugiperda* caterpillars in small arenas. *Heterorhabditis bacteriophora* (H.b.), *H. mexicana* (H.m.), *H. beicherriana* (H.be.), *H. zacatecana* (H.z.), *H. indica* (H.i.) and *H. ruandica* (H.r.), and *Steinernema feltiae* (S.f.), *Steinernema riobrave* (S.r.), *S. abbasi* and *S. carpocapsae* (S.c.). The mortality of individual caterpillars was evaluated over seven days post inoculation with ten infective juvenile nematodes per caterpillar. The commercial nematode *Steinernema carpocapsae* strain all was used as positive control. To facilitate readability, only a few highly virulent strains, which were selected for further tests, are highlighted in colors. The other strains are represented in grey. The dotted horizontal line represents 50% survival. Letters indicate significant differences (*p* < 0.05) obtained from multiple comparisons corrected for false discovery using the Benjamini and Hochberg method.

Mexican, Rwandan and commercial EPN species and strains differed in their speed at which they killed third-instar FAW caterpillars (χ^2^_(17)_ = 89, *p* < 0.001; Figure 4). The commercial *S. carpocapsae* strain All and *S. abbasi* were the fastest in killing caterpillars (*S. carpocapsae* All vs. *S. abbasi*: *p* > 0.05). Both strains killed 50% of the caterpillars within two days. The Rwandan *S. carpocapsae* RW14-G-R3a-2 was as fast as *S. abbasi* and reached LT_50_ after three days (*S. carpocapsae* RW14-G-R3a-2 vs. *S. abbasi*: *p* > 0.05). The fastest Mexican EPNs were *S. riobrave* MEX-15 (LT_50_ after four days) and *H. zacatecana* MEX-41 (50% mortality not reached after seven days). The Rwandan *H. ruandica* Rw18_M-Hr1a appeared slightly faster than the other strains of this species.

All tested EPN successfully reproduced in FAW caterpillars (Table S2), except for *Steinernema feltiae* and *Steinernema* sp. RW14-M-C2b-1, which killed too few caterpillars to evaluate their reproductivity.

### 3.3. Step 3: Assessing the Dose-Dependent Effectiveness of the Most Promising Entomopathogenic Nematodes against Different Stages of the Fall Armyworm

**Figure 5:**
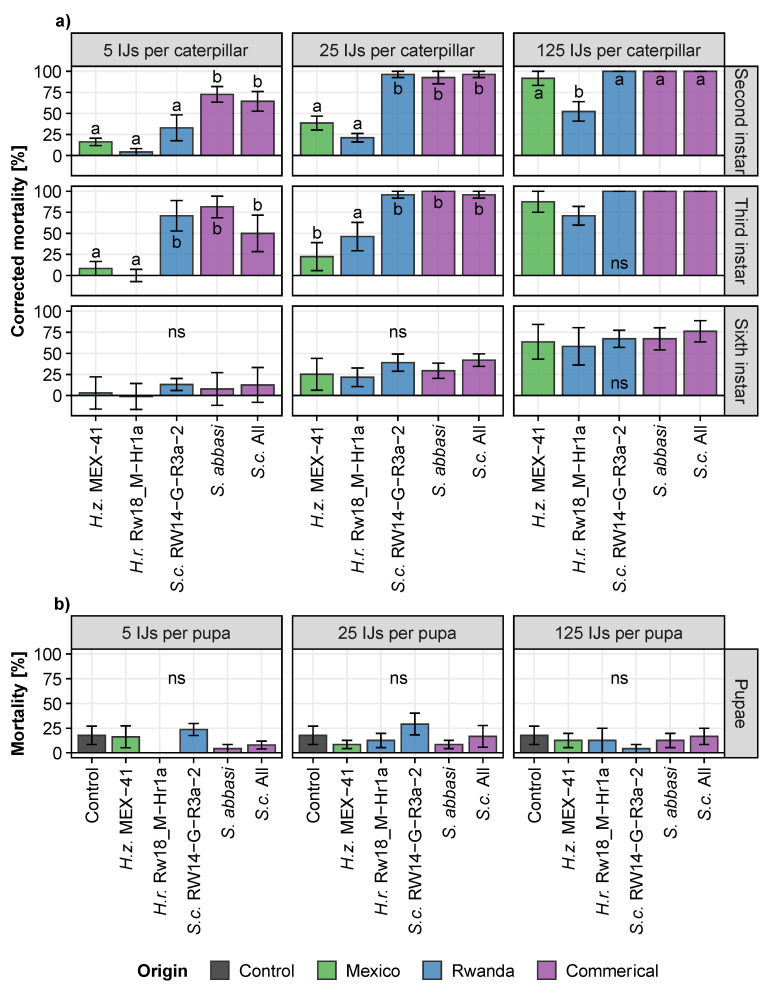
Virulence (mean ± SE) of the most promising Mexican, Rwandan and commercial entomopathogenic nematode strains on (**a**) second-, third- and sixth-instar caterpillars as well as on (**b**) pupae of *Spodoptera frugiperda* in small arenas. *H. zacatecana* (*H.z.*), *H. ruandica* (*H.r.*), *Steinernema abbasi* and *S. carpocapsae* (*S.c.*). Mortality was evaluated seven days post inoculation with either 5, 25 or 125 infective juvenile nematodes per caterpillar (**a**) or pupa (**b**). The virulence of each strain was compared to one another using multiple-comparisons corrected for false discovery using the Benjamini and Hochberg method. Letters indicate significant differences (*p* < 0.05) between treatments (ns = non-significant differences).

All EPN reduced FAW survival in dose-response experiments (χ^2^_(5)_ = 293, *p* < 0.001; Figure 5). FAW survival was different for different concentrations of EPN applied (χ^2^_(2)_ = 99, *p* < 0.001), as well as for the different FAW developmental stage (χ^2^_(3)_ = 63, *p* < 0.001). We found significant interactions between insect developmental stage and either EPN concentration or EPN treatment (Stage_Treatment χ^2^_(15)_ = 232, *p* < 0.001; Stage_Concentration: χ^2^_(6)_ = 60, *p* < 0.001).

Of all concentrations and FAW stages tested, the most virulent and equally effective EPN strains were the commercial *S. carpocapsae* All and *S. abbasi*, as well as the Rwandan *S. carpocapsae* RW14-G-R3a-2 (*p* > 0.05; Figure 5). The latter Rwandan strain was as effective as the two commercial strains (*p* > 0.05) on every development stage of FAW and at every tested concentration, except when five IJs were applied against second-instar caterpillars (*p* < 0.05; Figure 5). These strains were followed by *H. zacatecana* MEX-41 (*p* < 0.001). The Rwandan *H. ruandica* Rw18_M-Hr1a appeared to be the least virulent strain (Rw18_M-Hr1a vs. MEX-41: *p* < 0.05; Rw18_M-Hr1a vs. Steinermatid strains: *p* < 0.001). 

None of the tested EPN strains affected the survival of FAW pupae at the three tested concentrations of EPNs as compared to the control treatment with water only (*p* > 0.05; Figure 5b).

**Figure 6:**
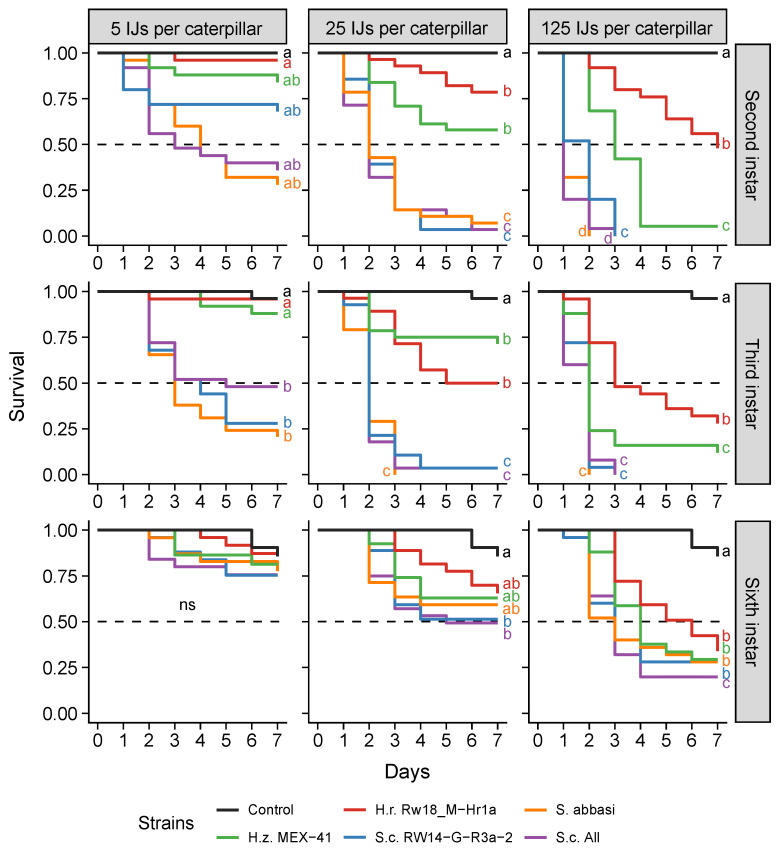
Killing speed of the most promising Mexican, Rwandan and commercial entomopathogenic nematode strains on second-, third- and sixth-instar caterpillars in small bioassay arenas. *H. zacatecana* (H.z.), *H. ruandica* (H.r.), *Steinernema abbasi* and *S. carpocapsae* (S.c.). The mortality of individual caterpillars was evaluated over seven days post inoculation with either 5, 25 or 125 infective juvenile nematodes per caterpillar. The dotted horizontal line represents a probability of 50% survival. Letters indicate significant differences (*p* < 0.05) obtained from multiple comparisons corrected for false discovery using the Benjamini and Hochberg method (ns = non-significant differences).

The killing speed of the most promising Rwandan, Mexican and commercial EPN differed significantly (χ^2^_(5)_ = 287, *p* < 0.001; Figure 6) and was dependent on EPN concentration (χ^2^_(2)_ = 156, *p* < 0.001), as well as the caterpillar developmental stage (χ^2^_(2)_ = 30, *p* < 0.001). Across all concentrations and stages tested, the fastest strains in killing FAW were the commercial strains *S. carpocapsae* All and *S. abbasi* as well as the Rwandan *S. carpocapsae* RW14-G-R3a-2 (*p* > 0.05; Figure 6). The Mexican *H. zacatecana* MEX-41 was slower (*p* < 0.001), and the Rwandan *H. ruandica* Rw18_M-Hr1a was the slowest EPN in killing FAW (Rw18_M-Hr1a vs. MEX-41: *p* < 0.05; Rw18_M-Hr1a vs. Steinermatid strains: *p* < 0.001).

At the highest concentration of EPN used (125 IJs per caterpillar), the three fastest EPNs (*S. carpocapsae* All, *S. abbasi* and *S. carpocapsae* RW14-G-R3a-2) killed 50% of either second- or third-instar caterpillars within one or two days (Figure 6). They needed three days to reach LT_50_ on sixth-instar caterpillars. The Mexican *H. zacatecana* MEX-41 killed 50% of second-, third- and sixth-instar caterpillars within three, two and four days, respectively, while the Rwandan *H. ruandica* Rw18_M-Hr1a needed seven, three and six days.

At the lowest concentration (5 IJs per caterpillar), the two commercial strains *S. carpocapsae* All and *S. abbasi* killed 50% of second-instar caterpillars within three or four days, respectively. None of the other nematodes caused more than 50% mortality of second-instar caterpillars within the seven-day exposure time. On third-instar caterpillars, the commercial strains *S. carpocapsae* All and *S. abbasi* as well as the Rwandan *S. carpocapsae* RW14-G-R3a-2 needed five, three and four days, respectively, to kill 50% of the caterpillars, while *H. ruandica* Rw18_M-Hr1a and *H. zacatecana* MEX-41 never reach 50% mortality. None of the EPN killed more than 50% of the sixth-instar caterpillars within seven days, when only five IJs were used.

All strains could reproduce in all FAW caterpillar stages tested, but not in pupae (Table S3). The dose of EPN used to inoculate the caterpillars as well as the insect stage at death, both positively affected EPN propagation (EPN dose: χ^2^_(2)_ = 845, *p* < 0.001; insect stage: χ^2^_(3)_ = 3959, *p* < 0.001). Notably, older larvae yielded more IJs than smaller ones per unit of weight. At a dose of five IJs per caterpillar, *S. carpocapsae* All and RW14-G-R3a-2 did not reproduce in sixth-instar caterpillars. At the same dose, the reproduction of *H. ruandica* strain Rw18_M-Hr1a in second- and third-instar caterpillars could not be evaluated as this nematode killed too few caterpillars. On average, a sixth-instar caterpillar (306 ± 89 mg at death [mean ± SD]) inoculated with 125 IJs of *H. ruandica, H. zacatecana*, *S. abassi* or *S. carpocapsae* produced 312′400 ± 131′249 (N = 15), 188′023 ± 117′192 (N = 15), 105′667 ± 644′424 (N = 18), 81′653 ± 62′120 (N = 36) IJs per larva (mean ± SD), respectively.

## 4. Discussion

We found that entomopathogenic nematodes (EPN) isolated from the invasive range of the fall armyworm (FAW) can be as effective in killing it as either the Mexican EPN that have likely co-evolved with FAW or as commercial EPN. For example, with a concentration as low as 25 nematodes per caterpillar, the Rwandan *Steinernema carpocapsae* RW14-G-R3a-2 was as virulent as the best performing Mexican and commercial EPN strains tested (Figure 5). This demonstrates that locally isolated EPN can be excellent candidates for the development of local biological control solutions against FAW. Although most EPN species are considered cosmopolitan, and therefore often do not require specific registration, the use of local strains may reduce potential risk of using genetically distinct alien strains or species as biocontrol agents in a target region.

In line with previous studies, we showed that EPN can be highly virulent against FAW caterpillars under laboratory conditions [17,18,19,39,40,41]. We further confirm here that the virulence of EPN on FAW varies importantly not only among species [17,22,40,41], but also among strains within a species [19]. For instance, we observed that *Heterorhabditis bacteriophora* strain MEX-17 was four times more virulent than *H. bacteriophora* MEX-33 (Figure 1). Similarly, Andalo et al. [17] reported that *Steinernema riobrave* was poorly effective against FAW, even at high concentrations (500 infective juveniles (IJs) per caterpillar), whereas we show that another *S. riobrave* was among the most effective strains in the present study (Figure 1, Figure 2 and Figure 3; strain MEX-15). The high variability in virulence between as well as within species indicates that thorough EPN screenings are essential before implementing biological control programs using EPN against FAW, and other insect pests.

The results show that the mortality of FAW caterpillars increases, as expected, with the concentration of EPN applied, but decreases with caterpillar age (instar), and that EPN cannot successfully infect pupae (Figure 5). It is known that pupae are often more resistant than other immature stages [42,43,44,45], but our results are inconsistent with Acharya et al. [22] who reported a 67% reduction in the emergence rate of FAW pupae after being exposed for five days to 600 IJs of *S. carpocapsae*. Similarly, Fuxa et al. [19] observed 20% mortality within three days of FAW pupae exposed to 40 IJs of *Steinernema feltiae*. We used either similar EPN concentrations [19] or exposure times and EPN species [22], implying that these discrepancies in pupal mortality are explained by the use of different EPN strains. However, for these previous studies it is not excluded that lingering EPN actually infected the emerging adults rather than the pupae. By dipping the pupae in a weak bleach solution before adult emergence, we eliminated this possibility. The infectiousness of EPN against adult lepidopteran has seldom been studied, but the few reports indicate that the insects are indeed susceptible to EPN at this stage [42,46,47]. 

With a concentration as low as 125 nematodes per caterpillar, the EPNs were found to be highly virulent (Figure 5). Such low, but effective concentrations are encouraging, as normally billions of EPN are applied per hectare in biological control programs [48,49]. It is realistic to envision that the application of just a few thousand EPN per maize plant suffices to significantly control FAW. Indeed, we have since found that the application of only 3000 EPNs per plant can be as effective as the use of a chemical insecticide (cypermethrin 5%) in controlling FAW under field conditions [50].

Effective FAW control not only demands highly virulent strains, but it will also require strains that are resistant to aboveground abiotic environmental factors. The EPN will have to be applied onto plant foliage in order to reach the caterpillars. In this aboveground environment EPN are exposed to, for them, unusually harsh and unfavorable conditions in terms of temperatures, UV light and risk of desiccation. Identifying highly infectious EPN strains that can tolerate these abiotic factors would further increase their potential as effective biocontrol agents against FAW under field conditions [51,52,53,54]. 

## 5. Conclusions

We show in this study that EPN isolated in the area of invasion of FAW can be as effective as area-of-origin or commercial EPNs to control FAW. The most virulent Rwandan EPN was *S. carpocapsae* RW14-G-R3a-2, which killed FAW caterpillars within just a few days (Figure 6). This killing power is a clear advantage over other biological control agents, such as entomopathogenic fungi, which usually need five to nine days to kill caterpillars and cannot actively search for their target [19,55,56,57]. Overall, we demonstrate that locally isolated EPN can be promising candidates for the biological control of FAW in invasive regions such as East Africa, and that our screening approach offers possibilities for developing countries to produce specific biocontrol products without relying on external sources.

## Figures and Tables

**Table 1:** Origin, strain, species and source of entomopathogenic nematodes.

Origin	Strain	Species (Authority)	Source
Commercial	NA (Dianem^®^)	*Heterorhabditis bacteriophora* (Poinar)	Provided by e-nema GmbH, Schwentinental, Germany
	NA	*Steinernema abbasi* (Elawad, Ahmad and Reid)	
	All (Nemastar^®^)	*Steinernema carpocapsae* (Weiser)	
	NA (Nemaplus^®^)	*Steinernema feltiae* (Filipjev)	
	H06 (HR-HB^®^)	*Heterorhabditis beicherriana* (Xing-Yue, Qi-Zhi, Nermut, Puza and Mracek)	Provided by Guangdong Academy of Sciences, China
	LN2 (HR-HI^®^)	*Heterorhabditis indica* (Poinar, Karunakar and David)	
Mexico	MEX-20	*Heterorhabditis atacamensis* (Edgington, Buddie, Moore, France, Merino and Hunt)	Collected by P. Bruno [28]
	MEX-14	*Heterorhabditis bacteriophora*	
	MEX-16		
	MEX-17		
	MEX-21		
	MEX-22		
	MEX-23		
	MEX-29		
	MEX-30		
	MEX-31		
	MEX-32		
	MEX-33		
	MEX-34		
	MEX-35		
	MEX-36		
	MEX-37		
	MEX-38		
	MEX-42		
	MEX-43		
	MEX-44		
	MEX-45		
	MEX-46		
	MEX-25	*Heterorhabditis mexicana* (Nguyen, Sharpiro-Ilan, Stuart, McCoy, James and Adams)	
	MEX-26		
	MEX-47		
	MEX-39	*Heterorhabditis zacatecana* (Machado, Bhat, Abolafia, Muller, Bruno, Fallet, Arce, Turlings, Bernal, Kajuga, Waweru and Toepfer)	
	MEX-40		
	MEX-41		
	MEX-15	*Steinernema riobrave* (Cabanillas, Poinar and Raulston)	
Rwanda	RW14-N-C4a	*Heterorhabditis ruandica* (Machado, Bhat, Abolafia, Muller, Bruno, Fallet, Arce, Turlings, Bernal, Kajuga, Waweru and Toepfer)	Provided by RAB, Rwanda [29]
	RW14-G-R3a-2	*Steinernema carpocapsae*	
	RW14-M-C2b-1	*Steinernema* sp. (closely related to *S. feltiae*)	
	Rw18_M-Hr1a	*Heterorhabditis ruandica*	Provided by RAB, Rwanda [30]
	Rw18_M-Hr1b		

## Data Availability

The data presented in this study are available on request from the corresponding author.

## Supplementary Materials

The following are available online at https://www.mdpi.com/article/10.3390/insects13020205/s1, Table S1: Reproduction of Mexican nematodes on third-instar fall armyworm caterpillars, Table S2: Reproduction of Rwandan, Mexican and commercial nematodes on third-instar fall armyworm caterpillars, Table S3: Reproduction of the most promising Rwandan, Mexican and commercial entomopathogenic nematode strains on second-, third- and sixth-instar caterpillars.
