# Supplementary material for: Comparative Screening of Mexican, Rwandan and Commercial Entomopathogenic Nematodes to Be Used against Invasive Fall Armyworm, Spodoptera frugiperda
Source: Insects. 2022 Feb 16;13(2):205. doi: 10.3390/insects13020205 (PMC8878727; doi:10.3390/insects13020205)
Supplement: Supplementary file 1 [file insects-13-00205-s001.zip › insects-1513620-supplementary.pdf]

Supplementary Table S1. Propagation of Mexican nematodes in fall armyworm caterpillars (mean ± SD). Individual third-instar caterpillars were exposed to ten infective juvenile nematodes in small arenas in laboratory. Reproduction was evaluated in nematode traps three weeks post mortality of the caterpillars in three independent experiments. The reproduction of strains that killed less than five caterpillars was not evaluated. Note: Nematodes killing speed varies importantly among strains (Figure 2). Hence, at the time of death, the caterpillars are not all at the same stage.

Species	Strain	IJs/mg ± SD	Number of cadavers
S. carpocapsae	All (Nemastar®)	372 ± 298.5	13
H. mexicana	MEX-26	361 ± 111.6	8
H. bacteriophora	MEX-30	333 ± 59.4	5
H. bacteriophora	MEX-36	327 ± 58.2	5
H. zacatecana	MEX-40	325 ± 80.6	6
H. bacteriophora	MEX-21	316 ± 95.7	11
H. bacteriophora	MEX-22	309 ± 99.4	7
H. bacteriophora	MEX-16	299 ± 48.8	7
H. zacatecana	MEX-41	297 ± 164.1	17
H. bacteriophora	MEX-43	294 ± 43.5	5
H. bacteriophora	MEX-37	291 ± 155.4	8
H. zacatecana	MEX-39	290 ± 184.2	10
H. bacteriophora	MEX-44	286 ± 52.9	7
H. bacteriophora	MEX-46	283 ± 134.7	9
H. mexicana	MEX-47	282 ± 159.1	12
H. bacteriophora	MEX-38	282 ± 64.8	8
H. bacteriophora	MEX-35	273 ± 73.3	11
H. atacamensis	MEX-20	272 ± 41.1	5
H. bacteriophora	MEX-34	259 ± 157.1	5
H. bacteriophora	MEX-31	249 ± 62.2	6
H. bacteriophora	MEX-17	249 ± 120.7	12
H. bacteriophora	MEX-42	232 ± 137.4	10
H. mexicana	MEX-25	227 ± 150.3	9
H. bacteriophora	MEX-32	212 ± 145.7	4
H. bacteriophora	MEX-14	211 ± 104.5	6
H. bacteriophora	MEX-45	202 ± 163.0	6
H. bacteriophora	MEX-29	200 ± 137.5	7
H. bacteriophora	MEX-23	186 ± 105.6	5
S. riobrave	MEX-15	119 ± 162.0	12

Supplementary Table S2. Reproduction of Mexican, Rwandan and commercial nematodes in fall armyworm caterpillars (mean ± SD). Individual third-instar caterpillars were exposed to ten infective juvenile nematodes in small arenas. Reproduction was evaluated in nematode traps three weeks post mortality of the caterpillars in three independent experiments. The reproduction of strains that killed less than five caterpillars was not evaluated. Note: Nematodes killing speed varies importantly among strains (Figure 4). Hence, at the time of death, the caterpillars are not all at the same stage.

Species	Strain	IJs/mg ± SD	Number of cadavers
H. indica	LN2 (HR-HI®)	426 ± 176.0	15
H. ruandica	Rw18_M-Hr1b	422 ± 85.4	12
H. ruandica	Rw18_M-Hr1a	349 ± 178.5	11
S. abbasi	NA	345 ± 212.3	12
H. zacatecana	MEX-41	337 ± 149.4	10
H. bacteriophora	MEX-17	310 ± 88.1	6
H. bacteriophora	NA (Dianem®)	303 ± 51.2	8
H. ruandica	RW14-N-C4a	290 ± 188.6	11
S. carpocapsae	All (Nemastar®)	280 ± 249.4	23
H. mexicana	MEX-47	246 ± 199.7	6
H. bacteriophora	MEX-35	237 ± 174.5	8
S. riobrave	MEX-15	176 ± 177.3	14
S. carpocapsae	RW14-G-R3a-2	171 ± 285.0	8

Supplementary Table S3. Propagation of the most promising Mexican, Rwandan and commercial entomopathogenic nematode strains (mean ± SD). Individual second-, third- or sixth-instar caterpillars were exposed to either 5, 25 or 125 infective juvenile nematodes in small arenas. Reproduction was evaluated in nematode traps three weeks post mortality of the caterpillars in three independent experiments. The reproduction of strains that killed less than five caterpillars was not evaluated (NA). Note: Nematodes killing speed varies importantly among strains (Figure 6). Hence, at the time of death, the caterpillars are not all the same stage.

**Inoculation of 2nd instar caterpillars**		**Propagation** EPN dose (IJs/arena)					
		**5**		**25**		**125**	
**Species**	**Strain**	**IJs/mg ± SD**	**N**	**IJs/mg ± SD**	**N**	**IJs/mg ± SD**	**N**
S. abbasi	NA	27 ± 70.5	17	247 ± 254.6	20	339 ± 293.4	19
S. carpocapsae	All (Nemastar®)	78 ± 153.5	16	288 ± 342.8	21	181 ± 244.0	19
H. zacatecana	MEX-41	240 ± 199.3	4	397 ± 234.5	7	268 ± 222.8	15
S. carpocapsae	RW14-G-R3a-2	205 ± 301.0	5	134 ± 197.2	19	250 ± 318.9	16
H. ruandica	Rw18_M-Hr1a	NA		187 ± 180.5	5	340 ± 177.3	11

**Inoculation of 3rd instar caterpillars**		**Propagation** EPN dose (IJs/arena)					
		**5**		**25**		**125**	
**Species**	**Strain**	**IJs/mg ± SD**	**N**	**IJs/mg ± SD**	**N**	**IJs/mg ± SD**	**N**
S. abbasi	NA	122 ± 172.4	21	253 ± 238.0	22	264 ± 205.0	21
S. carpocapsae	All (Nemastar®)	114 ± 178.1	11	165 ± 204.8	26	329 ± 227.2	25
H. zacatecana	MEX-41	NA		235 ± 201.5	7	217 ± 184.5	19
S. carpocapsae	RW14-G-R3a-2	48 ± 124.5	18	124 ± 175.9	24	308 ± 224.6	24
H. ruandica	Rw18_ M-Hr1a	NA		259 ± 174.6	12	366 ± 179.8	17

**Inoculation of 6th instar caterpillars**		**Propagation** EPN dose (IJs/arena)					
		**5**		**25**		**125**	
**Species**	**Strain**	**IJs/mg ± SD**	**N**	**IJs/mg ± SD**	**N**	**IJs/mg ± SD**	**N**
S. abbasi	NA	72 ± 150.9	5	305 ± 283.2	11	376 ± 219.3	18
S. carpocapsae	All (Nemastar®)	0 ± 1.2	6	168 ± 206.0	14	309 ± 154.4	20
H. zacatecana	MEX-41	614 ± 477.2	4	752 ± 278.9	10	582 ± 374.0	17
S. carpocapsae	RW14-G-R3a-2	0 ± 0.3	6	178 ± 231.1	13	252 ± 192.4	18
H. ruandica	Rw18_M-Hr1a	NA		732 ± 552.7	9	876 ± 435.8	16
